# Genomic Analysis and In Vitro Investigation of the Hop Resistance Phenotype of Two Novel *Loigolactobacillus backii* Strains, Isolated from Spoiled Beer

**DOI:** 10.3390/microorganisms11020280

**Published:** 2023-01-20

**Authors:** Despoina Eugenia Kiousi, Joanna Bucka-Kolendo, Adrian Wojtczak, Barbara Sokołowska, Agapi I. Doulgeraki, Alex Galanis

**Affiliations:** 1 Department of Molecular Biology and Genetics, Faculty of Health Sciences, Democritus University of Thrace, 68100 Alexandroupolis, Greece; 2 Culture Collection of Industrial Microorganisms, Microbiological Resources Center, Department of Microbiology, Prof. Waclaw Dabrowski Institute of Agricultural and Food Biotechnology, State Research Institute, Rakowiecka 36 Street, 02-532 Warsaw, Poland; 3 Department of Microbiology, Prof. Waclaw Dabrowski Institute of Agricultural and Food Biotechnology, State Research Institute, Rakowiecka 36 Street, 02-532 Warsaw, Poland; 4 Institute of Technology of Agricultural Products, Hellenic Agricultural Organization—DIMITRA, Sofokli Venizelou 1, 14123 Lycovrissi, Attica, Greece

**Keywords:** lactic acid bacteria, hop resistance, whole genome sequencing, spoiled beer, *Loigolactobacillus backii*

## Abstract

*Loigolactobacillus backii* is an important beer-spoiling species, exhibiting high hop tolerance. Here, we present the annotated whole genome sequence of two recently isolated strains, *Lg. backii* KKP 3565 and KKP 3566. Firstly, to study the genetic basis of the persistence of the two isolates in beer, a comprehensive bioinformatic analysis ensued. Their chromosome map was constructed, using whole-genome sequencing and assembly, revealing that the two strains carry genomes with a length of 2.79 Mb with a GC content of 40.68%. An average nucleotide identity (ANI) analysis demonstrated that the novel strains possess unique genomic sequences, also confirming their classification into the *Lg. backii* species. Their genome harbors numerous insertion sequences and plasmids, originating from other beer-spoiling species. Regarding their adaptation in brewery environment, homologous genes that confer resistance to hop were spotted, while the impact of hop bitters and pure beer on bacterial growth was investigated, in vitro. In brief, low hop concentrations were found to induce the proliferation of strains, while a higher concentration negatively affected their growth. Nonetheless, their ability to survive in pure beer indicated their tolerance to high hop concentrations. These results offer insight into the capacity of *Lg. backii* KKP 3566 and *Lg. backii* KKP 3566 to tolerate the extreme conditions prevalent in the brewery environment.

## 1. Introduction

In 2020, EU countries produced over 32 billion liters of beer, with Germany (24%) being the top producer, followed by Poland (12%), Spain (10%), Netherlands (8%), France (7%), Czech Rep. (6%) and Romania (5%) (EUROSTAT. Happy International Beer Day! Available online: https://ec.europa.eu/eurostat/web/products-eurostatnews/-/edn-20200807-1 (accessed on 5 September 2022)). Evidently, the brewing industry is an important branch of the food industry. It is known for centuries that hop components are responsible for the bitter taste of beer, also being recognized as a preservative [1]. Due to the evolution in the perception of taste and the association of bitterness with food hazard, the bitterness range has also changed, from the range of 20–60 IBU to 6–30 IBU in the case of lager. As a final product, the beer is considered a microbiologically stable beverage owing to the presence of alcohol, hop bitter compounds, high level of CO_2_, low level of O_2_ and low pH value [2,3]. However, lactic acid bacteria (LAB) can grow and spoil the beer, with *Levilactobacillus brevis* and *Pediococcus damnosus* being categorized as obligate spoilers found in most beer types. Other species, including *Fructilactobacillus lindneri*, *Loigolactobacillus backii*, *Secundilactobacillus paracollinoides* and *Furfurilactobacillus rossiae*, have been described as potential beer-spoiling bacteria [4,5]. As the ability of LAB to spoil beer is related to their tolerance to ethanol and acids, mainly iso-α-acids from hops which have an antibacterial effect [6], insight into the mechanism of resistance of LAB to hop compounds is necessary for understanding and estimating the level of risk involved in spoiling the final product. Some LAB strains are less sensitive to hop compounds and adapt well to the beer environment owing to the *horA*, *horC* and *hitA* genes, whereas the presence or absence of hop-resisting genes highly correlates with their beer-spoiling ability [7,8]. The *horA* gene encodes an ATP-dependent multidrug transporter that removes the hop bitter acids from the bacterial cells. This pump is located at the cytoplasmic membrane and has been found in many bacteria, e.g., *Lb. brevis* ABBC45, and was reported to be overexpressed when the strain has been exposed to hop compounds [7]. HorC is a proton motive force (PMF)-dependent multidrug transporter. In various LAB strains, *horC* is reported to be detected along with *horB*, which acts as a transcriptional repressor for *horC,* downregulating its expression in the hop-less bitter acids medium [3]. Accordingly, HitA participates in the transport of divalent cations, such as Mn^2+^, playing a significant role in maintaining the membrane pH gradient [3]. This is considered to help beer-spoiling LAB preserve cellular activities dependent on Mn^2+^, such as oxidative stress response, where HitA regulates the intracellular Mn^2+^ to minimize the stress induced by hop bitter acids [3]. Furthermore, beer-spoiling bacteria can also carry the fatty acid biosynthesis (FAS) gene cluster [3]. *FabZ*, that is coded by this cluster, is responsible for the production of the 3-hydroxyacyl-acyl-carrier-protein-dehydratase, catalyzing fatty acid synthesis de novo. These genes may provide diagnostic indicators to differentiate beer spoilage bacteria from non-spoilage ones.

Among beer-spoiling bacteria, little is known about the *Loigolactobacillus backii* species, which contains strains exhibiting high hop tolerance. Genetic and phenotypic characterization of *Lg. backii* is valuable to enhance the knowledge of their behavior, adaptation, and unique features, to design strategies for their control in the beverage industry. In this study, whole genome sequencing (WGS) and annotation, and in vitro analysis, were performed to determine the genetic and phenotypic characteristics of hop resistance of two, recently isolated, *Lg. backii* strains. More specifically, phylogenomic analysis and comparative genomics were used to study their spoiling characteristics, focusing on *horA*, *horC*, *hitA* and *fabZ* genes. The ability of the two novel strains to withstand increasing concentrations of hop bitters was also investigated, in vitro.

## 2. Materials and Methods

### 2.1. Bacterial Strains Isolation and Growth Conditions

Both strains of *Lg. backii* were isolated from lager beer (5.5% *v/v*) by ISO 15214:2000, as described previously by Bucka-Kolendo et al. [9]. They were cultured on MRS agar (DeMan, Rogosa, and Sharpe, Merck KGaA, Darmstadt, Germany) and UBA medium (Universal Beer Agar, Merck KGaA), and incubated at 30 °C for 3 to 5 days under anaerobic conditions.

### 2.2. DNA Extraction and Molecular Identification

Total bacterial DNA was extracted using the DNeasy PowerFood Microbial Kit (Qiagen, GmbH, Hilden, Germany), according to the manufacturer’s protocol. DNA purity was measured with the Nanodrop ND-1000 Spectrophotometer (Thermo Fisher Scientific, Watertown, MA, USA), and the concentration was quantified with Qubit 4.0 Fluorometer using the Qubit dsDNA BR Assay Kit (Invitrogen, Carlsbad, CA, USA). DNA samples were stored at −20 °C for further processing.

Strains were given the collection numbers KKP 3565 and KKP 3566 and deposited in the Culture Collection of Industrial Microorganisms—Microbiological Resource Center (IAFB, Warsaw, Poland). The 16S rDNA sequences of each strain were deposited in the GenBank NCBI database under the respective accession numbers OK2913330 for KKP 3565 and OK2873775 for KKP 3566. The genetic affiliation of the *Lg*. *backii* strains KKP 3565 and KKP 3566 were confirmed based on their phylogenetic analysis of the 16S rDNA and *pheS* sequences, as described previously by Bucka-Kolendo et al. [9,10] and by examining proteomic mass spectra profiles on MALDI-TOF MS [11].

### 2.3. Whole Genome Sequencing Analysis and De Novo Assembly

Genomic DNA was extracted from pure bacterial isolates using DNeasy PowerFood Microbial Kit (Qiagen) according to the manufacturer’s protocol. The DNA library was prepared using the Illumina DNA Prep kit (Illumina, San Diego, CA, USA) according to the manufacturer’s protocol (number #1000000025416v09). The magnetic bead normalization step was replaced with a manual normalization step, based on library concentration and average size as measured by the Qubit 4.0 Fluorometer with Qubit dsDNA HS Assay Kit (Thermo Fisher Scientific, Waltham, MA, USA) and the TapeStation 4200 Analyzer using the High Sensitivity D1000 ScreenTape Assay Kit (Agilent, Santa Clara, CA, USA), respectively. DNA was sequenced with a MiSeq next-generation sequencing platform, using the 2 × 151 bp paired-end MiSeq protocol and reagent v3 (600-cycle) kit (Illumina).

A total of 1,554,032 and 1,182,876 paired-end reads were obtained for *Lg. backii* KKP 3565 and KKP 3566, respectively. The quality of the reads was determined using FASTQC (v0.11.9) [12] and Trimmomatic was utilized to discard low-quality sequences (version 0.39) [13]. De novo assembly was executed with SPAdes and plasmid sequence extraction from the WGS with plasmidSPAdes (version 3.15.1) [14]. Scaffolding was performed with SSPACE [15]. Assembly metrics were calculated with the Quality Assessment Tool (QUAST, version 5.2.0) [16].

#### 2.3.1. Genome Annotation

Genome annotation was performed using Prokka (version 1.14.5) [17] and the local version of the Prokaryotic Genome Annotation Pipeline (PGAP) [18]. PlasmidFinder was utilized to detect the presence of plasmids in WGS [19]. Mobile genetic elements and prophage regions were investigated using MobileElementFinder [20] and PHAge Search Tool Enhanced Release (PHASTER) [21], respectively. ISFinder [22] was used to identify insertion sequence elements. Furthermore, for the detection of Clustered Regularly Interspaced Short Palindromic Repeats (CRISPR), arrays analysis with CRISPRDetect (version 2.4) [23] and PILER-CR [24] was performed. The presence of genes involved in antimicrobial resistance was determined using Resistance Gene Identifier (version 5.2.0) and ResFinder 4.1 [25,26]. The possibility of the novel strains being human pathogens was predicted using PathogenFinder 1.1 [27]. The EggNOGmapper (version 2.0) tool of the online EggNOG database (version 5.0) [28] was used for the classification of predicted proteins into Clusters of Orthologous Groups (COGs) and BlastKOALA (version 2.2) for the assignment of proteins into KEGG Orthology (KO) groups [29]. The CGview server [30] was utilized to visualize the whole genome sequence maps.

#### 2.3.2. Comparative Genomics

The genome sequences of all available *Lg. backii* strains, isolated from the brewery environment and spoiled beer (NCBI Genome. Available online: https://www.ncbi.nlm.nih.gov/genome/browse/#!/prokaryotes/45189/ (accessed on 1 October 2022)), (a total of 8 strains as of October 2022), were obtained using a python script. ANI was calculated with the python module Pyani (version 0.2.10) [31] and was used to verify strains’ uniqueness and taxonomic classification. Pangenome analysis of *Lg. backii* strains were performed with Roary (version 3.13.0) [32], and core genome sequences were used to construct an approximately-maximum-likelihood phylogenetic tree with FastTree 2.1 [33]. WGS of the available *Lg. backii* strains, three *Loigolactobacillus coryniformis*, four *Lactiplantibacillus plantarum*, and three *Lactiplantibacillus pentosus* strains were aligned with progressiveMauve [34]. Phylogenetic tree visualization was performed with the publicly-available online EMBL tool “Interactive Tree of Life” (iTol; version 6.1.1) [35].

#### 2.3.3. In Silico Investigation of Properties Related to Beer-Spoiling Capacity

Genes coding for proteins related to stress resistance were identified using annotation algorithms, including the KEGG database. Putative bacteriocin clusters were identified using BAGEL4 [36]. Comparative genomic analysis was used to predict the functionality of the annotated proteins involved in hop resistance. More specifically, Uniprot was searched for registered sequences of genes *horA*, *horC* and *hitA*, which were previously shown to be involved in the manifestation of hop resistance [3,37,38]. These sequences were queried against the WGS of the two novel strains. The alignment of sequences showing the higher sequence identity was performed with ClustalW [39]. Visualization of alignments was performed with Jalview [40]. Gene matrices for predicted proteins conferring resistance to stress and hop were constructed using GENE-E (GENE-E, Matrix visualization tool. Available online: https://software.broadinstitute.org/GENE-E/index.html (accessed on 1 November 2022)).

### 2.4. Determination of Lg. backii Strains Growth Inhibition in 5, 10, 20 and 30 IBU Hop Concentrations

The growth kinetics of *Lg. backii* KKP 3565 and *Lg. backii* KKP 3566 strains were estimated by maximum growth rate using the automated microbiology growth curve analysis system Bioscreen C Pro (Oy AB Ltd., Growth Curves, Finland), as described by Gientka et al. with modifications [41]. To determine the resistance to hop, modified MRS broth media with the bitterness of 5 IBU, 10 IBU, 20 IBU and 30 IBU (International Bitterness Units) were prepared by mixing concentrated MRS broth (Merck KGaA, Darmstadt, Germany), water, and beer (40 IBU) as shown in Table 1. An amount of 250 μL of the medium was applied to the wells, and 50 μL of 0.5 McF microbial culture in MRS broth was inoculated.

As a control, the strain’s ability to grow in pure beer was also tested. The beer chosen as the control was London Ale with 5.79% alcohol (*v*/*v*) and 43.6 IBU, which was used to prepare the starting concentration of 40 IBU beer. Beer used for evaluation was a mix of different hop compounds, mainly α-acids, iso-α-acids, xanthohumol, and iso-xanthohumol. All analyses of the beer were performed by standard methods of the European Brewery Convention (EBC) and Mitteleuropäische Brautechnische Analysenkommission (MEBAK).

In the following steps, beer adjusted to 40 IBU was used to achieve 5, 10, 20 and 30 IBU hop concentrations.

The Bioscreen analysis was performed for 72 h at 30 °C, and the OD600 was measured every hour. All assays were conducted in triplicates.

Based on the bacteria growth curves, the specific growth rate coefficients (μ) were determined from the equation:

(1) $$\mu =\frac{\ln OD_{max}-\ln OD_{min}}{t}$$

where ln *OD*_*max*_ is the natural logarithm of the value of the culture’s maximum optical density during the log phase, the ln *OD*_*min*_ is the natural logarithm of the value of the culture’s minimum optical density during the log phase, and *t* is the duration of the log phase [h].

### 2.5. Statistical Analysis

Data are presented as mean ± standard deviation (SD). Statistical analysis was performed using Statistica 14.0 (TIBCO Software, Palo Alto, CA, USA). The normality of the distribution was checked using the Shapiro–Wilk test. Equality of variance was studied using the Levene test and Brown–Forsythe test. To assess the significance of the influence of the examined factors, a one-way analysis of variance (ANOVA) was performed. HSD Tukey’s test was used after checking the assumptions to show differences between the groups.

## 3. Results and Discussion

### 3.1. Whole Genome Annotation and Gene Clustering

**Figure 1:**
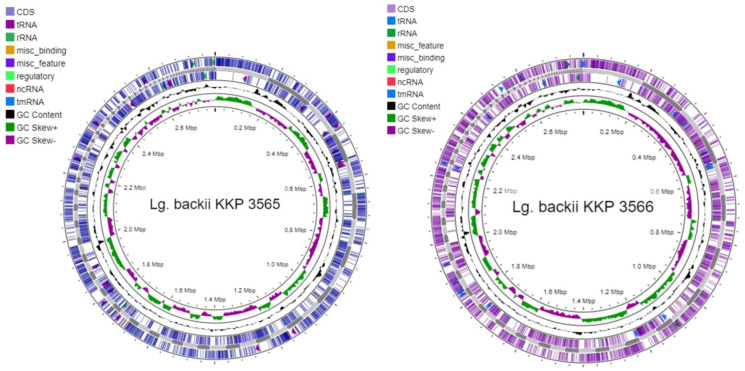
Whole genome sequence maps of *Lg. backii* KKP 3565 and KKP 3566 constructed with CGView.

Whole genome sequencing and assembly were performed to investigate the genomic features of the two novel strains *Lg*. *backii* KKP 3565 and *Lg*. *backii* KKP 3566. Both genomes carry a chromosome with a length of 2.79 Mb, with GC content of 40.68% (Table 2; Figure 1) and three plasmids (Table 3). These genome metrics are characteristic of the *Lg. backii* species that contain strains with a median genome length of 2.78 Mb and a median GC% content of 40.7% (NCBI Genome. Available online: https://www.ncbi.nlm.nih.gov/genome/?term=loigolactobacillus+backii (accessed on 1 November 2022)). Among the amended *Lactobacillus* genus, *Lg. backii* strains carry large chromosomes, evolved to allow survival in nutrient dense but diverse environments [42]. *Lg. backii* KKP 3565 and KKP 3566 code for 2640 and 2633 genes and for 2593 and 2582 CDSs, respectively. These genes cluster into 18 COG categories with known functions; the most represented group is amino acid metabolism and transport (E) followed by replication and repair (R) (Table 4). This classification of genes into clusters has been observed previously in members of the emended *Lactobacillus* genus. Of note, the high abundance of genes clustering in group E indicates the dependency of the strains for the extracellular supply of amino acids [43].

To investigate the genome stability of the strains, their WGS was searched for CRISPR arrays and mobile elements. Both strains lack CRISPR arrays and do not code for caspases, and thus they could be susceptible to phages and the incorporation of extrinsic DNA in their genome. CRISPR arrays are acquired with events of horizontal gene transfer between LAB and distant genera, as reflected in the distinctively different GC contents of these regions compared to the WGS [44]. Notably, phage immunity requires the synergy of complex processes to occur, and thus it could be mediated by alternative mechanisms [45]. However, both strains contain intact prophage sequences and a plethora of insertion and mobile elements, originating from other LAB, including *Lacticaseibacillus* spp, *Lactiplantibacillus* spp. and *Leuconostoc* spp. or commensal bacteria, such as *Fusobacterium nucleatus* (Table S1). Importantly, further genomic annotation of the mobile elements showed that the novel strains do not harbor transferable antimicrobial resistance genes, while prediction algorithms suggested the susceptibility of the two strains to common antibiotics (Table S2).

### 3.2. Phylogenomic and Pangenome Analysis 

**Figure 2:**
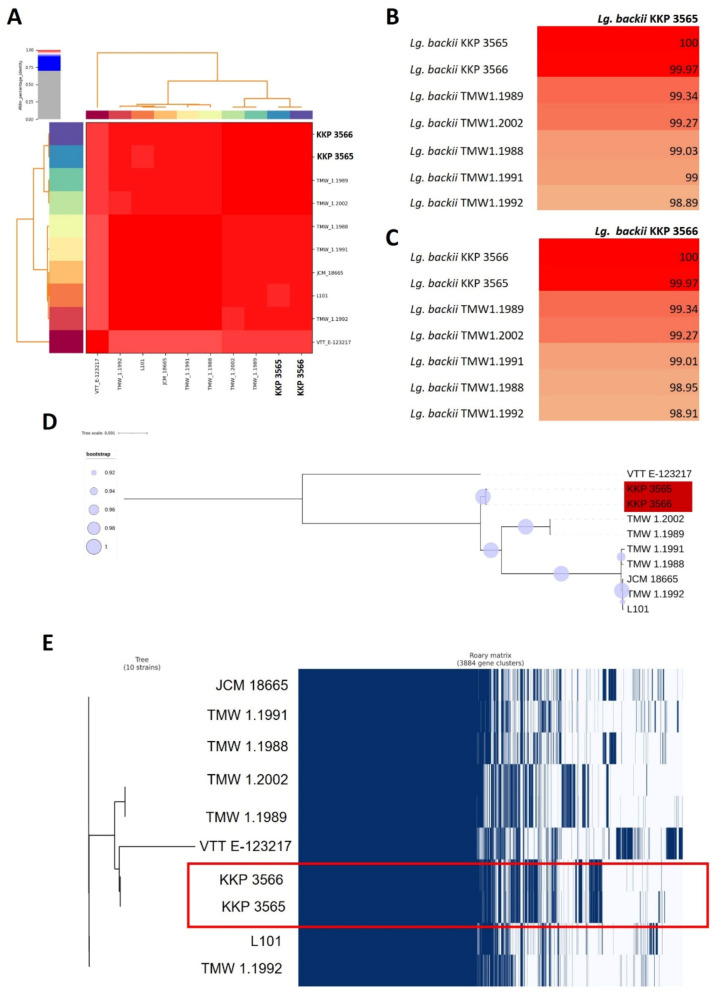
Phylogenomic and pangenome analysis of the two novel *Lg. backii* KKP 3565 and KKP 3566 strains. (**A**–**C**) ANI matrices of strains belonging to the *Lg. backii* species calculated by Pyani (version 0.2.10). (**D**) Approximately-maximum-likelihood phylogenetic tree of the core genome of *Lg. backii* strains. The visualization of the tree was performed with iTol. Highlighted in red are gene clusters contained in the genome of *Lg. backii* KKP 3565 and KKP 3566. (**E**) Presence/absence gene cluster of the *Lg. backii* pangenome. Enclosed in the red box are gene groups contained in the genome of the two strains of interest, *Lg. backii* KKP 3565 and KKP 3566.

ANI was calculated as a metric to infer phylogenetic relationships and strain uniqueness. It was shown that the two novel strains present ANI of 99.7%, presenting high similarity at the genetic level. Importantly, they share ANI of >98% with other members of the *Lg. backii* species, alluding to their correct taxonomic classification (ANI species cut off: 96%, Figure 2A–C) [46]. Furthermore, the WGS of the strains was aligned against members of the *Lg. backii* species and of other closely or more distantly-related LAB to produce a phylogenetic tree (Figure S1, Figure 2D). As shown in Figure S1, the two novel strains cluster with other members of the *Lg. backii* species, forming a distinct clade.

Pangenome analysis was utilized to detect core genome sequences of the species, as well as to pinpoint unique genetic loci in the genome of *Lg*. *backii* KKP 3565 and KKP 3566 (Figure 2E). The phylogenomic relationships of the strains based on the core genome of the *Lg. backii* species are depicted in Figure 2D. The core genome of the strains is dominated by proteins involved in replication and genetic information transport, as well as in carbohydrate metabolism (Table S3). Unique genomic sequences of the two strains were predicted using the same bioinformatic pipeline and the identified loci were annotated using eggNOG and Blastp. It was found that the unique protein groups are involved in chromosomal and plasmid replication (e.g., DNA topoisomerases, DNA primases and MobA/MobL mobilization proteins for plasmid transfer) and in genetic element transposition (e.g., IS family transposases) (Table S4). IS elements are widespread in LAB, playing an important role in their evolution and also contributing to adaptation in different environments [47]. Importantly, these elements are a key source of strain-specific genetic variability [48].

### 3.3. Comparative Genomic Analysis of Genes Related to Beer Spoilage and Adaptation to the Brewery Microenvironment

**Figure 3:**
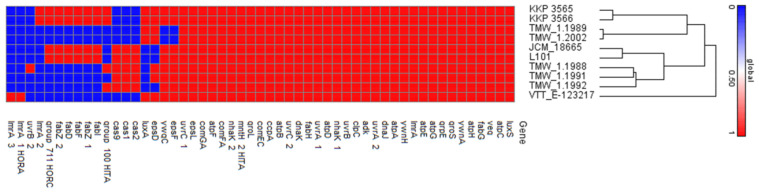
Gene matrix presenting CDS identified in the WGS of *Lg*. *backii* strains correlated with resistance to acid (e.g., F0-F1 ATPase), stress response and genome repair mechanisms (e.g., *dnaJ/K*, *uvrA*, *grpE*, *groS/L*), EPS production (e.g., *epsL/F*, *ywnA/H*), biofilm-related genes (e.g., *veg*, *comFC*, *comFA*, *comGA*, *comGB*, *comGC*, *luxS*) and hop resistance (*fabZ*, *hitA*, *horA/C*).

Although beer presents high microbiological stability, resisting to extrinsic contaminants due to its acidic pH, high concentration of hop bitters, alcohol, and low oxygen and nutrient content, LAB strains have successfully adapted to this hostile environment [38]. Growth of these bacteria in beer can increase turbidity, inducing a buttery odor and sourness due to the production of secondary metabolites and of exopolysaccharides. Comparative genomic studies of strains isolated from spoiled beer have aided in the identification of genes related to this phenotype [37]. In this sense, we sought to predict, in silico, genetic determinants involved in the spoilage capacity and ability of the strains to withstand stress relevant to the brewery environment (Figure 3). More specifically, their capability to produce diacetyl or lactic acid, two important secondary metabolites that contribute to beer spoilage was determined. Not surprisingly, a gene coding for an FMN-dependent L-lactate dehydrogenase (*lctO*) was annotated in both strains. The enzymatic activity of the product of this gene can vary, influencing matrix acidification levels. Additionally, a locus coding for a-acetolactate decarboxylase (*budA*) responsible for the production of a precursor (acetoin) of diacetyl was annotated, however diacetyl reductases were not found in the genome of the strains. Spontaneous non-enzymatic oxidative decarboxylation of acetoin for diacetyl formation may occur, however, diacetyl production is experimentally validated predominantly in beer spoiling *P. damnosus* strains [37]. Furthermore, we sought to determine the ability of the novel strains to produce biogenic amines, small compounds derived from amino acid decarboxylation or deamination that can have toxic implications for the consumer (e.g., nausea, headache, vomiting) [49]. Annotation algorithms did not provide evidence for the presence of enzymes involved in the production of these compounds.

Exopolysaccharide production can protect the cell from extrinsic stress, however, its accumulation in beverages results in higher, unfavorable viscosity [50]. To this end, genes involved in EPS biosynthesis and export were identified, namely *epsD*, *epsF*, *epsL*, *ywnqA* and *ywnqC*. EPS-producing strains present an enhanced ability to adhere to and colonize abiotic surfaces, ultimately leading to biofilm formation. Apart from EPS-synthesis related genes, other loci involved in biofilm formation were also identified in the genome of the strains. More specifically, a biofilm formation stimulator (veg family) is encoded by both strains, alongside members of the competence system (*comFC*, *comFA*, *comGA*, *comGB*, *comGC)* and the quorum sensing signal LuxS. Biofilm formation in the beverage industry can contribute to the deterioration of measuring organs or fermenters, causing significant economic loss [51]. Furthermore, we investigated the ability of the strains to produce antimicrobial compounds that could destabilize beer microbiota, further contributing to the spoilage phenotype. Analysis with BAGEL4 showed that the strains do not contain bacteriocin clusters. In this vein, no data exist for the presence of bacteriocin clusters in the genome of *Lg. backii* strains, although bacteriocin immunity proteins have been previously detected [37]. Indeed, bacteriocin immunity proteins, and more specifically, outer membrane porins and transporters, were annotated in the genome of both strains.

The ability of strains to withstand the hostile beer microenvironment was investigated in silico with the prediction of genes involved in acid, hop and extreme temperature resistance, as well as in protein folding and DNA repair (Figure 3). Indeed, a cluster for F0-F1ATPase proton pump (*atpABCDEFGH*) and a sodium–proton antiporter (*nhaK*) involved in acid tolerance were found in the genome of the strains. These genes are widespread in LAB strains adapted to acidified matrices, including potential probiotic strains, able to withstand the gastrointestinal conditions of the host [52]. Accordingly, genes coding for cold-shock proteins (*cspC*), members of the universal stress protein family (*uspA*) and of the UvrABC DNA damage system were annotated, among others, in the genome of the strains. Interestingly, analysis with ResFinder showed that both strains are resistant to heat treatments, due to the presence of the plasmid-encoded gene *clpL*. This gene was previously implicated in heat resistance of *Listeria monocytogenes* [53], while also being possibly involved in penicillin resistance [54].

**Figure 4:**
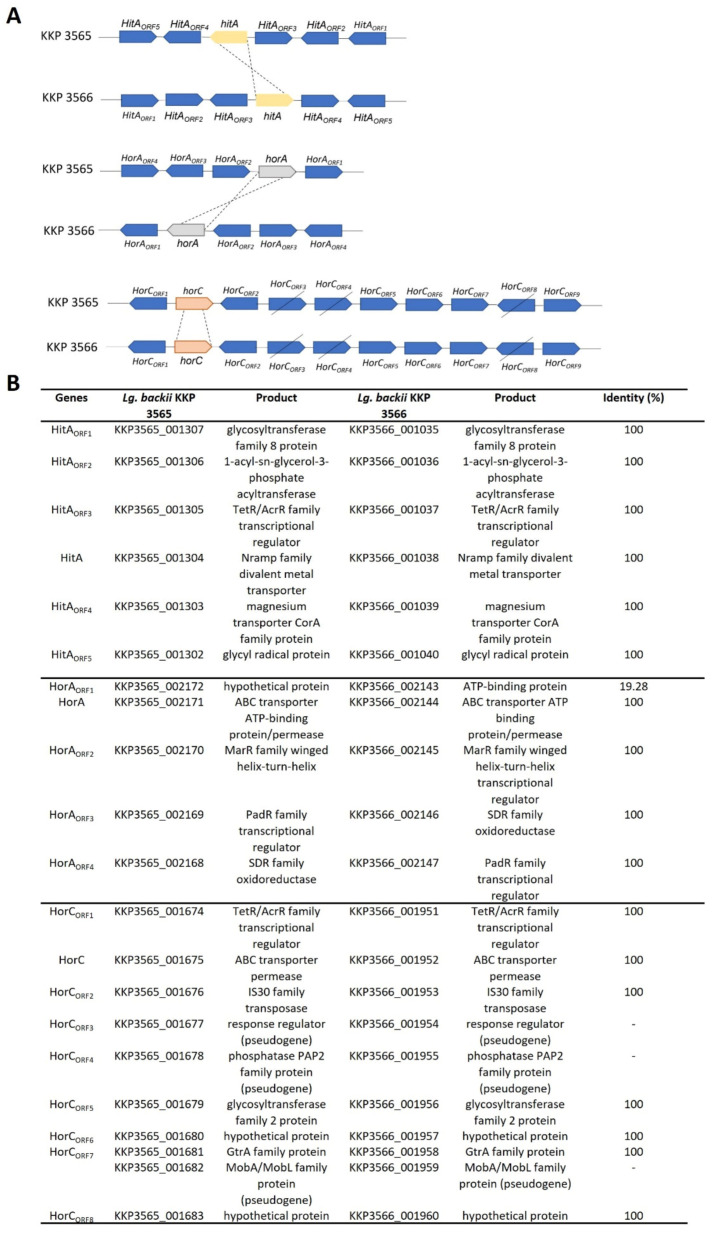
Cluster analysis of the hop-resistant genes *horA*, *horC* and *hitA* annotated in the genome of *Lg*. *backii* KKP 3565 and KKP 3566. (**A**) Schematic representation of the *horA*, *horC* and *hitA* clusters contained in the genome of the two novel strains. (**B**) Description and pairwise comparison of genes (percentage identity) contained in the three clusters of *Lg*. *backii* KKP 3565 and KKP 3566.

Next, we determined the ability of the two strains to code for proteins involved in hop resistance. Hop bitters possess antimicrobial properties, limiting the growth of contaminants in beer [38]. Comparative genomic studies of beer-spoiling species have highlighted that HorA, an ABC-type multidrug transporter, HorC, a PMF-dependent multidrug transporter, and HitA, a divalent metal cation transporter, show a strong correlation with the resistant phenotype [55]. Furthermore, FabZ (3-hydroxyacyl-[acyl-carrier-protein] dehydratase), an enzyme involved in fatty acid biosynthesis has been additionally proposed as a diagnostic marker for beer-spoiling species. In this vein, we managed to identify and pinpoint the location of the genes in the WGS of both strains, using annotation algorithms and local Blastp (Figure 4). Homologous genes presenting sequence identity and structural conservation to sequences derived from beer-spoiling *L. brevis* strains were identified in the genome of the novel strains, as shown in Table 5. Cluster analysis showed that their structure is conserved (Figure 4), showing similarities to those previously described in beer-spoiling species [8]. Among these genes, *horC* resides in plasmid sequences and not in the chromosome of the strains. In this context, plasmids of both novel strains carry *fabZ* among other genes involved in fatty acid biosynthesis (i.e., *fabH*, *fabD*, *fabF*, *fabI*). The presence of genes conferring resistance to heat stress and hop in plasmid sequences supports the transfer of these elements in other microbes inhabiting the beer microenvironment [8]. Indeed, we showed that the plasmids carried by the two strains originate from other beer-inhabiting *Lg. backii* and *L. brevis* strains (Table 3). In agreement to this, strains that exhibit the hop-resistant phenotype do not present phylogenetic closeness, therefore supporting the transmission of these elements between strains that inhabit the same matrix [56].

### 3.4. Beer-Spoilage Ability of Lg. backii KKP3565 and KKP3566 Strains

**Figure 5:**
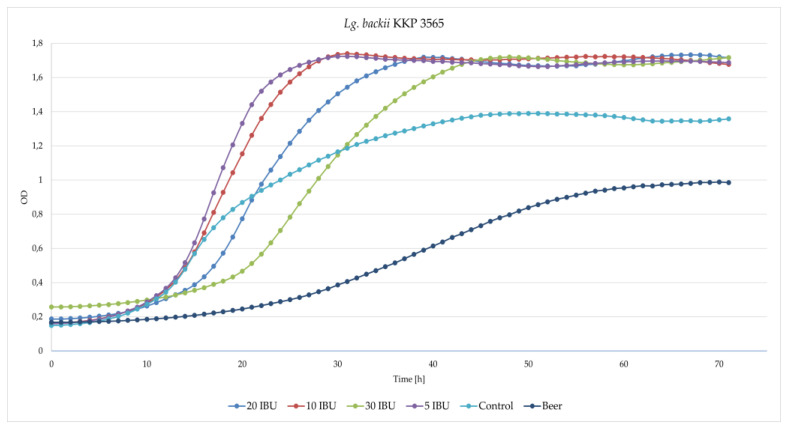
Growth curves of the strain *Lg. backii* KKP 3565 in MRS media containing various hop concentrations.

**Figure 6:**
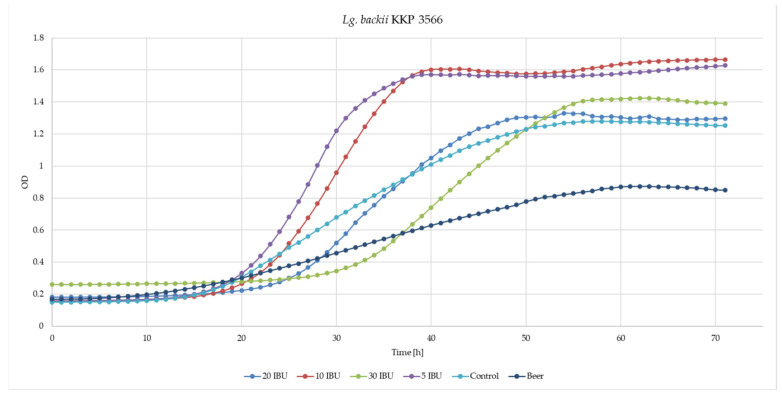
Growth curves of the strain *Lg. backii* KKP 3566 in MRS media containing various hop concentrations.

A modified medium containing different concentrations of hop bitters (Table 1) was used to assess the capability of *Lg*. *backii* KKP 3565 and KKP 3566 strains to grow. Both strains demonstrated the ability to grow in MRS broth enriched with beer and in pure beer (Figure 5 and Figure 6, respectively). The *Lg*. *backii* KKP 3565 showed slightly higher growth parameters [both the specific growth rate (µ) and the maximum optical density (OD_max_)] in all tested media than *Lg*. *backii* KKP 3566 (Table 6 and Table 7, respectively).

*Lg. backii* KKP 3565 demonstrated better growth on the beer-enriched medium than on the control one, except for the 30 IBU variant, where the growth dynamic was worse than that of the control. On the other hand, variants in 5 IBU, 10 IBU and 20 IBU showed better growth dynamics compared to the control. *Lg*. *backii* KKP 3565 not only showed resistance to the ingredients contained in beer, but lower concentrations of beer even stimulated its growth. *Lg*. *backii* KKP 3566 showed similar growth on beer-enriched MRS broth with higher concentration of hop bitters (20 IBU and 30 IBU) than on MRS broth, and better expansion in the lower concentration (5 IBU and 10 IBU). Only 5 IBU showed better growth dynamics than the control, so a small addition of beer stimulates its growth. *Lg*. *backii* KKP 3566 showed resistance to the ingredients contained in beer, but not as much as *Lg*. *backii* KKP 3565. In addition, preliminary data showed that both *Lg. backii* strains exhibited better adaptation to hop than other analyzed strains isolated from beers, including *L*. *brevis* strains (data not shown). The prevalence of the iso-α-acids in the used beer could affect the lower antibacterial activity and impact the lower growth inhibition of the strains.

## 4. Conclusions

Two novel strains, *Lg. backii* KKP 3565 and KKP 3566 previously isolated from spoiled beer, were characterized in this study. Utilizing in silico and in vitro analyses, we sought to investigate their persistence in spoiled beer. Comparative genomic analysis within the *Lg. backii* species, and with other beer-spoilage bacteria, highlighted the presence of shared genes involved in the adaptation to the brewery environment and stress tolerance mechanisms. In this context, homologous genes (*hitA*, *horA*, and *horC*) conferring resistance to hop were pinpointed in the genome of the novel strains. Furthermore, both strains were able to survive in pure beer and tolerate different hop concentrations, in vitro, suggesting adaptation to the extreme conditions prevalent in the brewery environment. Further studies will provide a better insight into the contribution of the identified loci in the manifestation of the beer spoiling phenotype.

## Figures and Tables

**Table 1:** Scheme of the studied bitterness concentrations.

	5 IBU	10 IBU	20 IBU	30 IBU	Beer 43.6 IBU	Control
MRS broth concentrate (2×)	50%	50%	50%	-	-	50%
MRS broth concentrate (4×)	-	-	-	25%	-	-
Water	37,5%	25%	-		-	50%
Beer (40 IBU)	12,5%	25%	50%	75%	-	-
Beer (43.6 IBU)	-	-	-	-	100%	-

**Table 2:** Genomic features of *Lg*. *backii* KKP 3565 and KKP 3566.

Element	*Lg. backii* KKP 3565	*Lg. backii* KKP 3566
Length	2,796,657 bp	2,798,617 bp
GC content (%)	40.68	40.68
Genes (total)	2640	2633
CDSs	2593	2582
tRNAs	39	42
ncRNAs	4	4
Pseudogenes	47	50
Cas arrays	0	0
Insertion Elements	124	175
Mobile Elements	19	20
Prophages		
Intact	2	2
Incomplete	1	0
Questionable	0	0
Bacteriocin Production	No	No
Bacteriocin Immunity	Yes	Yes
Pathogenicity (%)	0.106	0.099

**Table 3:** Plasmids contained in *Lg. backii* KKP 3565 and KKP 3566.

Strain	Plasmid AN	PlasmidFinder Annotation	Origin	Identity
*Lg. backii* KKP 3565	NZ_CP014896.1	rep28_2_LBPp6g007 (LBPp6)	*Lg. backi* TMW 1.1992	0.99928
	NZ_CP031183.1	rep38_1_rep (pLBUC03)	*Lv. brevis* UCCLB95	0.99447
	NZ_CP014889.1	rep38_1_rep (pLBUC03)	*Lg. backii* TMW 1.1991	0.99145
*Lg. backii* KKP 3566	NZ_CP014896.1	rep28_2_LBPp6g007 (LBPp6)	*Lg. backii* TMW 1.1992	0.99918
	NZ_CP031183.1	rep38_1_rep (pLBUC03),	*Lv. brevis* UCCLB95	0.99345
	NZ_CP014889.1	rep38_1_rep (pLBUC03)	*Lg. backii* TMW 1.1991	0.99048

**Table 4:** Categorization of *Lg. backii* KKP 3565 and *Lg. backii* KKP 3566 CDS in COGS.

COG	*Lg. backii* KKP 3565	*Lg. backii* KKP 3566
C—Energy production and conversion	95 (4.3%)	95 (4.26%)
D—Cell cycle control and mitosis	46 (2.07%)	47 (2.11%)
E—Amino acid metabolism and transport	203 (9.13%)	204 (9.14%)
F—Nucleotide metabolism and transport	102 (4.58%)	107 (4.8%)
G—Carbohydrate metabolism and transport	128 (5.75%)	130 (5.82%)
H—Coenzyme metabolism	89 (4%)	90 (4.03%)
I—Lipid metabolism	64 (2.88%)	64 (2.87%)
J—Translation	173 (7.78%)	173 (7.75%)
K—Transcription	176 (7.92%)	177 (7.93%)
L—Replication and repair	195 (8.77%)	195 (8.74%)
M—Cell wall/membrane/envelop biogenesis	136 (6.12%)	133 (5.96%)
N—Cell motility	9 (0.4%)	9 (0.4%)
O—Post-translational modification, protein turnover, chaperone functions	49 (2.2%)	49 (2.2%)
P—Inorganic ion transport and metabolism	129 (5.8%)	131 (5.87%)
Q—Secondary structure	24 (1.08%)	24 (1.08%)
T—Signal transduction	47 (2.11%)	47 (2.11%)
U—Intracellular trafficking and secretion	52 (2.34%)	52 (2.34%)
V—Defense mechanisms	34 (1.53%)	34 (1.52%)
S—Function unknown	472 (21.23%)	471 (21.1%)
Total	2223 (100%)	2232 (100%)

**Table 5:** Sequence identity of genes conferring hop resistance carried by the novel strains with genes identified in beer-spoiling species.

Gene	Function	Locus Tag (*Lg. backii)*	Reference (AN)	Identity (%)	E-Value
*hitA*	Nramp family divalent metal transporter	KKP3565_001038	J7LK56_LEVBR	83	0.0
*hitA*	Nramp family divalent metal transporter	KKP3566_001304	J7LK56_LEVBR	83	0.0
*horA*	ABC transporter ATP binding protein/permease	KKP3565_002144	O32748_LEVBR	97	0.0
*horA*	ABC transporter ATP binding protein/permease	KKP3566_002171	O32748_LEVBR	97	0.0
*horC*	ABC transporter permease	KKP3565_001952	Q6I7K2_LEVBR	95	0.0
*horC*	ABC transporter permease	KKP3566_001675	Q6I7K2_LEVBR	95	0.0

**Table 6:** Growth dynamic (µ) of *Lg*. *backii* KKP 3565 and KKP 3566 in different hop concentrations using Tukey’s HSD test (α = 0.05). Lowercase—significant differences between the media for a given strain, and Uppercase—significant differences between strains on a given medium.

µ	Control	5 IBU	10 IBU	20 IBU	30 IBU	Beer 43.6 IBU
KKP 3565	0.05013 ± 0.0002 cB	0.0829 ± 0.0003 eB	0.0825 ± 0.0005 eB	0.0607 ± 0.0002 dB	0.0437 ± 0.0003 bB	0.0304 ± 0.0005 aB
KKP 3566	0.0475 ± 0.0018 dA	0.0629 ± 0.0008 eA	0.0429 ± 0.0010 cA	0.0420 ± 0.0024 cA	0.0318 ± 0.0006 bA	0.0280 ± 0.0005 aA

**Table 7:** Maximum optical density (OD max) of *Lg*. *backii* KKP 3565 and KKP 3566 in different hop concentrations using Tukey’s HSD test (α = 0.05). Lowercase—significant differences between the media for a given strain, and Uppercase—significant differences between strains on a given medium.

OD max	Control	5 IBU	10 IBU	20 IBU	30 IBU	Beer 43.6 IBU
KKP 3565	1.3833 ± 0.0223 bB	1.7240 ± 0.0210 cB	1.7398 ± 0.0021 cB	1.7190 ± 0.0055 cB	1.7125 ± 0.0839 cB	0.9855 ± 0.0217 aB
KKP 3566	1.2438 ± 0.0945 bA	1.5725 ± 0.0414 cdA	1.6420 ± 0.0810 dA	1.3298 ± 0.1482 bA	1.4190 ± 0.0508 bcA	0.8495 ± 0.0133 aA

## Data Availability

The *Loigolactobacillus backii* strain KKP3565 and *Loigolactobacillus backii* strain KKP3566 genome sequence has been deposited at DDBJ/ENA/GenBank under the accessions JAPTYS000000000 and JAPTYT000000000, respectively. The versions described in this paper are JAPTYS010000000 and JAPTYT010000000, respectively.

## Supplementary Materials

The following supporting information can be downloaded at: https://www.mdpi.com/article/10.3390/microorganisms11020280/s1, Figure S1: Phylogenetic tree of *Lg. backii* strains and of other closely or distantly-related LAB based on their WGS. WGS alignment was performed using progressive Mauve and tree visualization using the iTol server; Table S1: Prophage regions and insertion elements annotated in the genome of *Lg. backii* KKP 3565 and KKP 3566 using PHASTER and ISFinder, respectively; Table S2: Resistance phenotypes of *Lg. backii* KKP 3565 and KKP 3566 predicted by ResFinder; Table S3: Annotation of core genome sequences of the *Lg. backii* species using EggNOG; Table S4: Annotation of unique genome sequences of *Lg. backii* KKP 3565 and KKP 3566 derived from pangenome analysis.
