# Supplementary figures and images for: Genomic Analysis and In Vitro Investigation of the Hop Resistance Phenotype of Two Novel Loigolactobacillus backii Strains, Isolated from Spoiled Beer
Source: Microorganisms. 2023 Jan 20;11(2):280. doi: 10.3390/microorganisms11020280 (PMC9967799; doi:10.3390/microorganisms11020280)

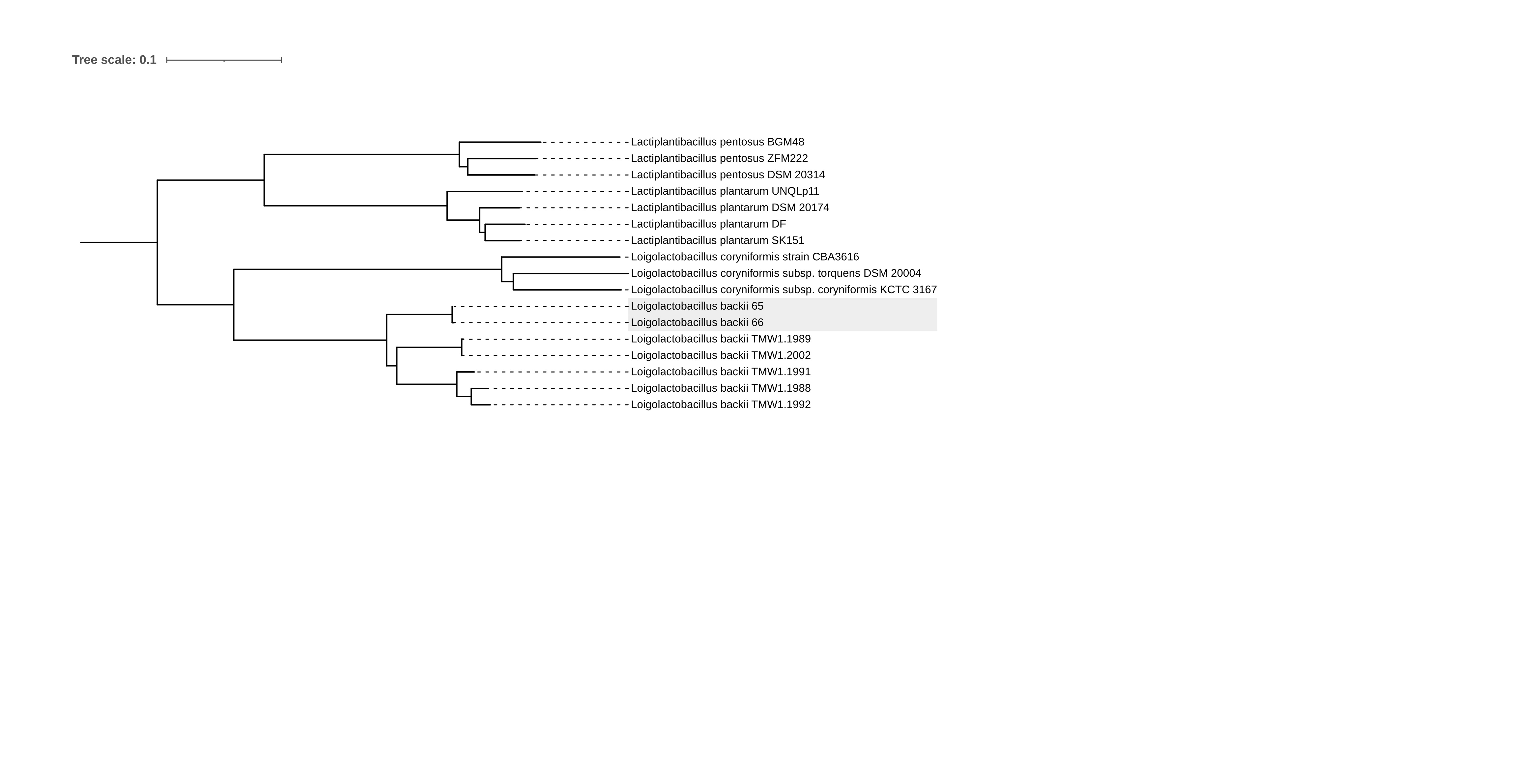

Supplement: Supplementary file 1 [file microorganisms-11-00280-s001.zip › Supplementary Figure 1.jpg]
